# Analysis of Birth Weights of a Rural Hospital

**DOI:** 10.4103/0970-0218.66879

**Published:** 2010-04

**Authors:** Shyam V Ashtekar, Madhav B Kulkarni, Vaishali S Sadavarte, Ratna S Ashtekar

**Affiliations:** Yashwantrao Chavan Maharashtra Open University, Gangapur, Maharashtra, India; 1 Department of Mathematics and Statistics, BYK College of Commerce, Nashik, Maharashtra, India; 2 Department of Biotechnology, HPT RYK College, Nashik, Maharashtra, India; 3 Private Hospital in Dindori, Nashik, Maharashtra, India

**Keywords:** Age of mother, birth order, birth weight, gender

## Abstract

**Background:**

Low birth weight remains a major reason behind childhood malnutrition. The NFHS findings show no dent in this problem.

**Objective:**

This study was undertaken to explore change in birth weights in a period from 1989 to 2007 and any associations thereof.

**Materials and Methods:**

All birth records of a private rural hospital spanning two decades (1989-2007) were analyzed for birth weight, age of mother, gender, birth order of the baby, proportion of pre-term babies and low birth weight babies.

**Results:**

No change was observed in the average birth weights (average 2.71 kg) over the period. Although the birth weight shows some expected variance with the age of mother, it was found to have no relation with the baby’s birth order and gender. The low birth weight proportion is about 24% and shows little difference before and after the series midpoint of year 1998.

**Conclusion:**

The birth weights have hardly changed in this population in the two decades.

## Introduction

Birth weight is a major determinant of child’s health and nutrition. In India, birth weight has remained low, with the NFHS reported proportion of low birth weight (LBW) babies about 23% for rural and 19% for urban population.(1) The proportion of LBW has improved only marginally from NFHS 1(2) and 2 rounds.(3) However, NFHS data does not offer quantitative estimates of birth weights. The NFHS 3 reports association of low birth weight to place of residence (urban or rural), age of mother, religion and caste, birth order of the baby, education, wealth and use of tobacco. NFHS 3 reports LBW for Maharashtra state to be 22%.(4) However, internet search for birth weight studies from India yields very little literature, some of which is cited below.

A study from urban population of Bhilai reports LBW proportion of 23% which is rather high for urban population.(5). A study from South India using primary data from Ambedkar district in Tamil Nadu reports that the mean birth weights (2.77 kg) have shown a very marginal improvement (70 g) in the period from 1969 to 1993.(6). It has been observed from the same study that low birth weight (<2.5 kg) proportion reduced significantly from 27.2% to 15.9% in rural and 19.1% to 10.8% in urban areas over the decades. In a hospital-based study in Kolkota (2005), the proportion of LBW was 34% with a mean BW of 2.64 kg.(7) The proportion of low birth weight babies in a Kerala study (1997) was found to be 18%.(8)

Systematic studies with standardized methods are rare in Indian literature. In this situation, secondary data from hospitals and gram panchayat birth records can be highly rewarding, though the issue of standardization of weighing scales and methodology in recording of birth weights are bound to remain a concern. Also the large proportion of home births create huge gaps in birth weight information, mainly as they are likely to be from disadvantaged communities and hence from the lower end of the birth weight distribution. We took up this study with an impression that the proportion of low birth weight babies must be declining over the two decades at least in hospital data. The secondary data available to us from this rural hospital should provide some insights.

## Materials and Methods

This retrospective study is an analysis of the childbirth records of a private nursing home in Dindori block of district Nashik (Maharashtra) from 1989 to 2007. This block of Nashik district has a mixed population of tribal and non-tribal communities. The block has both prospering grape growing villages as well as tribal communities migrating in search of seasonal labour after one paddy crop in monsoon. The block has two rural hospitals, 10 primary health centers (PHC) and 66 health sub-centers. The block has 95 doctors (private and public sector) distributed in clusters.(9) The block has 393 Anganwadis and good transport infrastructure. Jeeps, cars and autos have now nearly replaced bullock carts for patient transport. Road traffic has apparently grown many times in the two decades. Mobiles are ubiquitous in the last five years even in tribal areas. The economy has visibly progressed.

The hospital in this study was started in 1988 with a view to offer rational ethical care to rural people. The Obstetric-Gynecology work dominated clinical work in all these years. This study includes all the 2586 births conducted in the hospital in this period. The families reported from several villages (14 to 133) in the two decades and had both tribal and non-tribal families. The case records were available in year wise bound books. Incomplete case records were deleted. Factors such as weight, mother’s education, and hemoglobin were not mentioned on the birth record because they were recorded on the ANC (Ante Natal Care) record sheet, which was not available for this retrospective study. The baby weighing machines reportedly changed three times in the study period, but the same type and brand was used (weighing pan with spring balance). There was no conscious attempt for standardization or validation of the scale; hence instrument error cannot be eliminated. The observer remained nearly the same for most births except in the last five years when one more doctor joined the hospital. The spot location of the scale has also remained unchanged for the two decades-in the delivery room, ensuring the same eye level from the delivery table. The practice of weighing the baby after wrapping in old cotton sari-cloth adds about 25 g to the bare baby weight, (we found that two such clothes together weighed 50 g).

## Results

The data covers period from 1989 to 2007 -19 calendar years. The number of births recorded in the first four years in the hospital has been clubbed since the numbers were small as the hospital was new in the locality and home births were the norm. The work picked up from 1993 onwards, with a maximum of 202 births in 2003. The expected annual number of deliveries in the block (2.64 lakh general population in 2001) is 6600+ at a birth rate 25. Other deliveries took place at other hospitals including the PHCs and Rural Hospitals and at homes. The data (2586 births) represents about 2% of the childbirth cases in the block over the period of 19 years.

**Figure 1:**
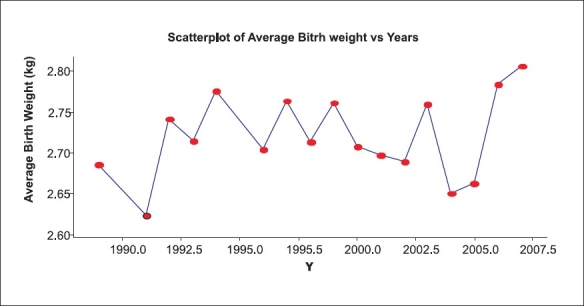
Average BW as time series

**Figure 2:**
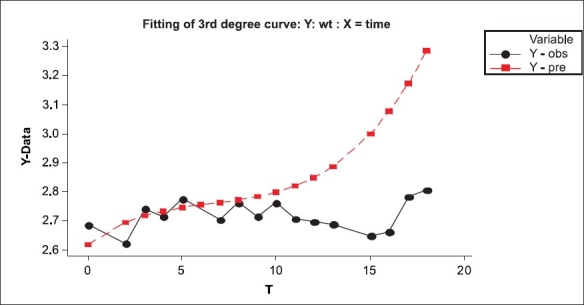
Regression of average BW on time series

The average BW is generally influenced by, among other things, proportion of pre-term babies. The data shows a variable proportion of pre-term deliveries (3% in 2002 to 15% in 1995) with an average level of 6% [Table 1]. Total number of twin births was 16 in the entire series. Each of the twins has been entered separately for birth weight. The average birth weight, in our data, is 2.71 kg with an SD of birth weight 0.48 kg [Table 2, and Figure 1], which remains unchanged through the period with some exceptions. The data on birth weight of babies are available year-wise. This can be looked upon as a time series or one can make use of regression analysis where average birth weight (Y) is regressed on year (X). Since the number of children in each year is different based on which averages are worked out, the weighted regression analysis is appropriate. (Here ‘weight’ being number of children in that year.) In this analysis, year is on X scale and average BW on Y scale. The number of children in each age year varies from 1989 to 2007 [Table 3]. We have fitted linear, quadratic and cubic polynomial to this data. This takes care of non-linear nature of data as seen in scatter plot [Figure 1], and the regression is shown in Figure 2. The analysis is shown below:

**Table 1:** Percentage of pre-term babies

Year	Pre-term	Total	Percentage
1989-1992	17	225	7.55
1993	10	100	10.00
1994	10	165	6.06
1995	21	132	15.91
1996	12	145	8.28
1997	11	179	6.15
1998	8	194	4.12
1999	6	159	3.77
2000	8	197	4.06
2001	16	173	9.25
2002	5	164	3.05
2003	4	203	1.97
2004	14	184	7.61
2005-2007	17	366	4.65

Total	159	2586	6.15

**Table 2:** Distribution of BW over two decades

Year	No of cases	Mean birth weight (kg)	SD of birth weight(kg)
1989-1992	208	2.63	0.47
1993	95	2.71	0.47
1994	158	2.78	0.44
1995	123	2.65	0.53
1996	137	2.7	0.54
1997	173	173	0.52
1998	188	2.71	0.45
1999	155	2.76	0.47
2000	192	2.71	0.49
2001	167	2.7	0.45
2002	155	2.69	0.45
2003	202	2.76	0.47
2004	180	2.65	0.53
2005	183	2.66	0.46
2006	142	2.78	0.43
2007	40	2.81	0.40

Total	2498	2.71	0.48

**Table 3:** Percentage of LBW in last two decades

Year	%LBW	Year	%LBW
1989-92	25.48	2000	26.56
1993	23.16	2001	25.15
1994	18.99	2002	26.45
1995	31.71	2003	21.29
1996	34.31	2004	26.67
1997	17.34	2005	24.04
1998	26.06	2006	20.42
1999	19.35	2007	17.5
		Pooled (1989-2007)	24.22

The regression equation is

The regression equation is

Mean birth weight=2.62 + 0.0479 T1-0.00598 T2 + 0.000208 T3

**Table:** 

(Data on 17 years used),					
Predictor	Coef	SE Coef		T	P
Constant	2.6188	0.06331		41.36	0.000
T1	0.0479	0.02526		1.90	0.080
T2	-0.00598	0.00306		-1.95	0.073
T3	0.000208	0.00011		1.91	0.078
S=0.536387, R-Sq=22.7%					
Analysis of variance					
Source	DF	SS	MS	F	P
Regression	3	1.0978	0.3659	1.27	0.325
Residual Error	13	3.7402	0.2877		
Total	16	4.8380			
Source	DF	Seq SS			
T1	1	0.0001			
T2	1	0.0448			

Value of R^2^ is poor. It indicates even the third degree curve is also not a good fit. (We performed analysis in the presence and absence of two suspected outliers – data for year 2 and 14). Since this fit is not ‘good’ and all the regression coefficients are not significant, it supports the claim that birth weight has all most remained the same although there are some unexplained outliers. We conclude that the average birth weight has remained nearly stagnant over the study period. There is no gender difference in the average birth weight. The t-test for testing equality of means shows that the difference in mean birth weight for male (*N*=1335, mean BW 2.72 kg) and female babies (N=1114, mean BW 2.70 kg) is not significant (*P* value >0.05).

The overall proportion of low birth weight (<2.5 kg) babies in the period (2498 cases including pre-term babies) was 24%, with some unexplained spikes in the year 1995 and 1996.

The year 1998 is midpoint of the series. Incidentally an ultrasound machine was introduced in service in 1998 to improve ANC diagnosis. When tested for BW difference in the series before and after 1998; there was slight difference in proportion of low birth weight (LBW) babies before and after 1998 (25% and 24%) which was statistically not significant (*P* value >0.05).

The data on age of mother were available for 1947 cases. Only 2% of the mothers were below 18 years of age. The mention of age on the case paper is approximate and may not be very factual, as often happens in rural areas and more so when a woman arrives for childbirth in labor pains. The mean BW for this data is 2.71 kg. The average BW does not change across different age groups of mother [Table 4]. BW is also not significantly different between age groups, mothers below (BW 2.71 kg) and above 21 years (BW 2.72 kg). There is no difference in mean BW of babies of mothers below or above 18 years of age (*P* value 0.86). The correlation coefficient between age of a mother and birth weight of baby was weak (0.159), though statistically significant.

**Table 4:** Distribution of BW according to age of mother

Age Group(in Yrs)	No of cases	Mean BW (kg)	SD of BW (kg)
Below 18	36	2.67	0.5
18+	1233	2.72	0.47
23+	515	2.72	0.47
29+	163	2.68	0.51

Total	1947	2.71	0.47

## Discussion

Birth weight is a major determinant of childhood malnutrition and mortality. Low birth weight (LBW) is a hard-core factor in chronic malnutrition in India as seen from NFHS3 data. While post-natal efforts for improving child’s weight are important, improving the BW itself is still a major issue. NFHS data shows(1–4) that birth weight is influenced by many socio-economic factors like being urban or rural, education, birth order, tobacco use by mother, wealth, religion and caste. These factors act through proximate factors such as a) age of mother, b) nutrition of the mother including BMI and Hemoglobin, c) quality of ante-natal care (ANC) affecting fetal nutrition, d) spacing of pregnancies, e) order of birth, etc.

This study, with its limitations of possible instrument error, offers a cross-section of rural birth weights over the last twenty years in a single block of Maharashtra. Notably, the data shows that the LBW proportion and mean birth weights have changed little over the years. The NFHS surveys show that LBW percentage among weighed children showed very small decline through the decade viz 24.7 (1992-1993), 23.9 (1998-1999) and 23.3 (2005-2006). The present study is therefore consistent with the NFHS data.

This study shows some expected positive correlation of BW with age of mother. This reinforces the general importance of preventing early marriages and childbirths. The NFHS3 findings of 2003-2004(4) estimate the proportion of under-age mothers at 18.3% for rural Maharashtra. Though this study shows a very small percentage of mothers below 18 years (2%), it must be noted that the age data is missing from nearly 25% (642 out of 2586 cases) of the case records. The missing age records may be about teenage mothers.

This study shows no influence of gender of the baby on BW. This, against the new understanding on gender differences on birth weight (mean for boys 3.46 kg and mean for girls 3.23 kg) in WHO Growth standards,(10) may be because of the overall low positioning of this sample in global distribution of birth weights. However, this difference may manifest only at optimal conditions. The order of birth also makes no difference to birth weight in this study.

Did the ANC make a difference to the BW? This is a difficult question to answer from our data. This is because we have no access to records of ANC visits. We also do not know how many of these women were registered for ANC with PHCs. Many unregistered cases need to be admitted for delivery in this hospital. We assume that the data is homogenous for hemoglobin (Hb) since many rural women have a starting level Hb under 8 g and rural women’s weights do not vary highly. The proportion of low body mass index (BMI) for rural women in Maharashtra in NFHS3 is as high as 43%.(4)

## Conclusion

Birth weights have not changed much in the study period in this rural block despite the changing economy of the region. We need to act on the proximate social-familial and gender factors for improving birth weights.

## References

[1] NFHS3, IIPS Child Health. http://www.nfhsindia.org/factsheet.html.

[2] NFHS1, IIPS Maternal and Child Health. http://www.nfhsindia.org.

[3] NFHS2, IIPS State findings (key Indicators). http://www.nfhsindia.org.

[4] NFHS3 IIPS Child Health. http://www.nfhsindia.org/factsheet.html.

[5] Misra M, Mishra S, Sharadamma (1995). Epidemiology of low birth weight in an industrial area in India. J Trop Pediatr.

[6] Antonisamy B, Rao PS, Sivaram M (1994). Changing scenario of birth weight in South India. Indian Pediatr.

[7] Bisai S, Mahalanabis D, Sen A, Bose K, Datta N (2007). Maternal early second trimester pregnancy weight in relation to birth outcome among Bengalee Hindus of Kolkata. India. Ann Hum Biol.

[8] Radhakrishnan T, Thankappan KR, Sarma PS Socio-economic and demographic factors associated with birth weight: Results of a community based study in Kerala (1997), India. Achutha Menon Centre for Health Science Studies, Trivandrum, India.

[9] Ashtekar S, Mankad D (2006). PC Sen Best Paper Award (2002). Profile of care providers in five blocks of Nasik Maharashtra: A window on rural health services in Maharashtra. Indian J Public Health.

[10] The WHO Child Growth Standards, Weight-for-age boys and girls birth to 13 weeks (z-scores). http://www.who.int/childgrowth/standards/en/.

